# Parental asthma and childhood asthma phenotypes: maternal associations with persistent asthma

**DOI:** 10.1136/bmjresp-2026-004245

**Published:** 2026-08-04

**Authors:** Marianne Rørholt Grefslie, Siri Eldevik Håberg, Maria C Magnus, Tone K Omsland, Per Magnus

**Affiliations:** 1 Centre for Fertility and Health, Norwegian Institute of Public Health, Oslo, Norway; 2 Department of Paediatric Medicine, Oslo University Hospital, Oslo, Norway; 3 University of Bergen Department of Global Public Health and Primary Care, Bergen, Norway; 4 University of Oslo Department of Community Medicine and Global Health, Oslo, Norway

**Keywords:** Asthma Epidemiology, Paediatric asthma, Asthma

## Abstract

**Background:**

Maternal asthma has been more strongly associated with asthma in offspring than paternal asthma, but the influence of parental asthma on distinct asthma phenotypes remains unclear.

**Methods:**

We studied 16 055 children from the Norwegian Mother, Father and Child Cohort Study (MoBa). Parental asthma was reported at recruitment and classified as no parental asthma (reference), maternal asthma only, paternal asthma only or both parents with asthma. Offspring asthma was assessed at ages 3, 7 and 14 years through questionnaire-based parental reports, and four phenotypes were defined: never asthma, early-onset transient, early-onset persistent and late-onset asthma. Logistic regression was used to estimate associations between parental asthma status and offspring phenotypes, adjusting for sociodemographic, lifestyle and atopic factors, including atopic eczema and pollen/hay fever.

**Results:**

Offspring asthma phenotypes were distributed as follows: never asthma 88.8%, early-onset transient asthma 3.3%, early-onset persistent asthma 2.7% and late-onset asthma 5.2%. Maternal asthma was associated with increased odds of all offspring asthma phenotypes: early-onset transient asthma (OR 2.09, 95% CI 1.52 to 2.88), early-onset persistent asthma (OR 3.47, 95% CI 2.56 to 4.71) and late-onset asthma (OR 2.18, 95% CI 1.69 to 2.81). Paternal asthma showed similar associations, although point estimates were generally smaller: early-onset transient asthma (OR 2.01, 95% CI 1.48 to 2.73), early-onset persistent asthma (OR 2.49, 95% CI 1.81 to 3.44) and late-onset asthma (OR 1.85, 95% CI 1.44 to 2.38). Asthma in both parents was most strongly associated with early-onset persistent asthma (OR 5.34, 95% CI 2.86 to 9.98), whereas associations with early-onset transient and late-onset asthma were smaller and accompanied by wider CIs. The overall pattern of associations was similar across analyses stratified by parental atopy.

**Conclusions:**

Associations with maternal asthma were more pronounced than those with paternal asthma, particularly for early-onset persistent and late-onset phenotypes, although CIs overlapped for most comparisons. In contrast, early-onset transient asthma showed less differentiation by parental asthma status.

**WHAT IS ALREADY KNOWN ON THIS TOPIC**

- Maternal asthma is associated with increased risk of offspring asthma in early childhood more than paternal asthma, but phenotype-specific familial risk is poorly understood.

**WHAT THIS STUDY ADDS**

- Our findings indicate a greater association with maternal than paternal asthma, especially for early-onset persistent asthma. The maternal effect persists regardless of parental atopy. Early-onset transient asthma appears less influenced by familial risk and may reflect viral wheeze.

**HOW THIS STUDY MIGHT AFFECT RESEARCH, PRACTICE OR POLICY**

- Family history, particularly maternal asthma, may help identify children at increased risk of persistent asthma. Recognition of phenotype-specific familial patterns may help identify children at increased risk of persistent asthma and inform future approaches to monitoring and risk stratification.

## Introduction

 Asthma is the most common chronic disease in children, affecting approximately 9% of children and 11% of adolescents worldwide.^1^ While prevalence rates are higher in high-income countries, mortality is markedly greater in low- and middle-income countries.^2^ Moreover, lower socioeconomic status is an important risk factor for asthma even within high-income settings, including the United States.^2^ Childhood asthma is of particular importance due to its potential persistence into adolescence and adulthood, as well as its substantial healthcare burden.^3 4^

Parental asthma is a well-established risk factor for asthma in offspring. Several studies suggest that maternal asthma confers a stronger risk than paternal asthma, potentially due to in utero exposures and maternal immune factors.^5–8^ However, many studies relied on single time point assessments, and did not differentiate between longitudinal asthma phenotypes, limiting understanding of phenotype-specific familial risk.^9 10^ Few studies have repeated asthma assessments across childhood and adolescence to derive longitudinal asthma phenotypes. This study examines whether maternal and paternal asthma and parental atopy are associated with distinct childhood asthma phenotypes (early-onset transient, early-onset persistent and late-onset asthma) and whether these associations differ between maternal and paternal asthma.

Asthma is a heterogeneous condition with distinct phenotypes, including early-onset transient, early-onset persistent and late-onset asthma.^11^ These phenotypes differ in timing, severity, persistence and likely underlying mechanisms. Early-onset transient asthma has been associated with environmental exposures, including respiratory infections, whereas early-onset persistent and late-onset asthma are more strongly associated with allergic sensitisation and atopy, in addition to environmental influences.^7 12 13^ Parental asthma may influence these phenotypes differently; for example, maternal asthma may affect the in utero environment and foetal immune development, potentially contributing to persistent or late-onset allergic phenotypes.^13^ Accordingly, familial susceptibility may play a greater role in early-onset persistent asthma than in transient wheezing phenotypes.^14^ Improved understanding of these relationships may provide insights into familial risk patterns and mechanisms underlying childhood asthma heterogeneity.

Using data from the Norwegian Mother, Father and Child Cohort Study, MoBa,^15^ we investigated associations between parental asthma and childhood asthma phenotypes. Parental asthma was reported at recruitment, and offspring asthma was assessed longitudinally through questionnaires at ages 3, 7 and 14 years. We hypothesised that parental asthma, particularly maternal asthma, would be more strongly associated with persistent than transient asthma phenotypes, given previous evidence that maternal asthma confers a higher risk of offspring asthma than paternal asthma^16^ and that persistent asthma is more strongly linked to familial and atopic susceptibility than transient wheezing phenotypes.^17 18^

## Methods

### Study population

We used data from the Norwegian Mother, Father and Child Cohort Study, MoBa,^15^ a population-based pregnancy cohort conducted by the Norwegian Institute of Public Health. Pregnant women were recruited nationwide between 1999 and 2008 at approximately 15–18 weeks of gestation. The participation rate among invited women was 41%. Partners were invited concurrently.^15^ Women could participate with more than one pregnancy, resulting in the recruitment of ~95 000 mothers, ~75 500 fathers and ~1 14 500 children.^19^ Written informed consent was obtained from all participants, and MoBa is regulated by the Norwegian Health Registry Act.

**Figure 1:**
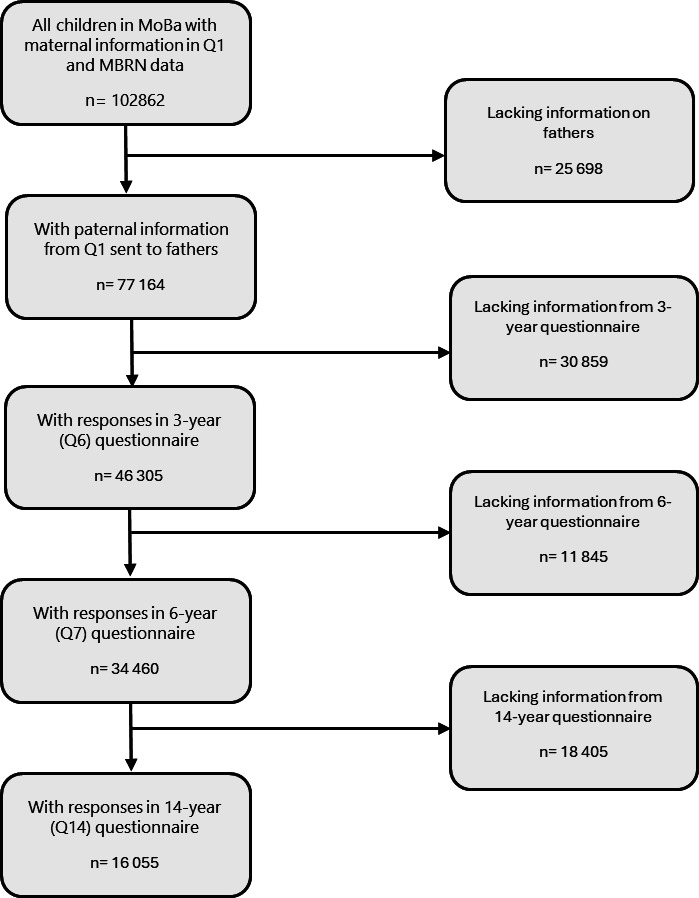
Flowchart of study participant selection. Number of children included in the analysis from the Norwegian Mother, Father and Child Cohort Study (MoBa) after applying inclusion criteria and availability of parental and child questionnaire data. Q1, baseline questionnaire at ~ week 15 of pregnancy; Q6, 3-year follow-up; Q7, 7-year follow-up; Q14, 14-year follow-up.

MoBa data were linked to the Medical Birth Registry of Norway (MBRN)^20^ using unique personal identification numbers. Parents completed comprehensive questionnaires during pregnancy and at several time points after delivery. We included all children with available information on asthma status at ages 3, 7 and 14 years, together with parental data from baseline questionnaires and MBRN. Because questionnaire participation declined across follow-up—46 302 at age 3, 42 025 at age 7 and 21 927 at age 14—and our aim was to study longitudinal asthma phenotypes, we restricted analyses to children with completed questionnaires at all three follow-up ages. A flow diagram of inclusion and attrition from the full MoBa cohort to the analytic sample is presented in figure 1, and baseline characteristics of the eligible cohort and the analytic sample are shown in online supplemental table 1). After these exclusions, the total study population comprised 16 055 children (figure 1). The study was approved by the Regional Committees for Medical and Health Research Ethics (approval no. 184620).

### Patient and public involvement

Participants and the public were not involved in the development, execution, or reporting of this study. This research used secondary data from the Norwegian Mother, Father and Child Cohort Study (MoBa), which collects information through questionnaires and linkage to national registries.

### Parental asthma

Parental asthma was defined as self-reported asthma up to recruitment. Maternal asthma was defined as asthma reported before or during the index pregnancy and paternal asthma as self-reported history of asthma (response options: ‘has now’ or ‘has had’). Both measures therefore reflect asthma history prior to recruitment, although ascertained using slightly different question formats.

Participants were categorised into four mutually exclusive exposure groups: no parental asthma (reference), maternal asthma only, paternal asthma only or both parents with asthma. Analyses were restricted to children with complete information on both maternal and paternal asthma to ensure consistent classification of parental exposure groups.

Information on parental asthma severity, treatment and current activity was not available. Age at onset was collected only for fathers and was not included in the analyses. Parental asthma was self-reported and was not independently validated against clinical records or registry data.

### Childhood asthma phenotypes

Childhood asthma was assessed via maternal report at ages 3, 7 and 14. At each follow-up, mothers were asked whether the child had experienced asthma as a long-term health condition (no; yes, currently; or yes, previously). Based on reported asthma status across follow-up, four mutually exclusive phenotypes were defined as follows:

- *Never asthma:* no asthma reported at any age.
- *Early-onset transient asthma:* asthma at age 3 but not at ages 7 or 14.
- *Early-onset persistent asthma:* asthma at age 3 and at least once at ages 7 or 14.
- *Late-onset asthma:* no asthma at age 3, but asthma at ages 7 and/or 14.

In sensitivity analyses, we applied a stricter definition of persistent asthma, requiring asthma to be reported at 7 and 14 years, irrespective of asthma status at age 3 years, and re-estimated the associations with parental asthma.

Children were classified as non-asthmatic if mothers explicitly denied asthma at all time points. Children were also classified as non-asthmatic if asthma responses were missing while other questionnaire items were completed, because unanswered asthma items in otherwise completed questionnaires were considered unlikely to reflect current asthma. Responses indicating previous but not current asthma were not classified as current asthma at that follow-up, as phenotypes were defined by asthma status at specific ages (3, 7 and 14 years). Asthma occurring between assessments could not be reliably assigned to predefined phenotype categories.

To assess whether these classification decisions influenced the results, we repeated the analyses restricting the sample to children with explicit yes/no asthma responses at all follow-up ages (3, 7 and 14 years), excluding children with missing asthma information at any time point.

Objective clinical measures were unavailable; however, maternal reports of childhood asthma in MoBa have previously been validated against dispensed asthma medications recorded in the Norwegian Prescription Database (NorPD). In a subset of 2056 children, maternal report of antiasthmatic medication use showed a sensitivity of 85% and specificity of 97% compared with dispensed prescriptions recorded in the NorPD. Maternal report of asthma showed a sensitivity of 80%, and only 1.2% of children without reported asthma had dispensed medications.^21^

### Covariates and potential confounders

Analyses included potential confounders plausibly associated with both parental and offspring asthma (online supplemental figure 1). These comprised maternal age at delivery (<25, 25–29, 30–34, 35–39, ≥40 years), maternal and paternal education (9-year elementary school, 1–3 years of high school, ≥4 years of university/college), pre-pregnancy BMI for both parents (continuous, mean±SD) and maternal and paternal smoking during pregnancy (yes/no).

Maternal and paternal atopy (yes/no) were included as potential confounders and indicators of familial allergic predisposition, given their association with asthma risk in both parents and offspring. This included a history of atopic eczema, pollen/hay fever, or both, either current or past. The term ‘pollen/hay fever’ reflects questionnaire wording and corresponds to seasonal allergic rhinitis rather than perennial forms. However, as atopy may lie on the causal pathway between parental asthma and offspring asthma, adjustment could result in over-adjustment. Therefore, we additionally assessed models excluding parental atopy from the adjustment set to evaluate the robustness of the findings. birth weight (mean±SD) was considered a potential mediator and not adjusted for, whereas offspring sex was treated as a potential effect modifier and not adjusted for.

Maternal and paternal characteristics were obtained from parent-reported questionnaires, while parental age at delivery, offspring sex and birth weight were obtained from the Medical Birth Registry of Norway (MBRN).

### Follow-up and attrition

Participation declined across follow-up. Attrition was primarily associated with sociodemographic factors such as parental education and smoking. Maternal asthma prevalence remained stable across participation groups (7.6% in the full cohort, 7.4% at age 3 follow-up, 7.3% at age 7 follow-up and 7.4% at age 14 follow-up), suggesting limited evidence of substantial differential attrition according to maternal asthma status. Missingness was largely due to questionnaire non-response rather than incomplete covariate data; therefore, multiple imputation was not applied. Baseline characteristics of the eligible cohort and analytic sample are presented in online supplemental table 1) to assess potential selection bias, and participant flow is shown in figure 1.

### Statistical analysis

The prevalence of each childhood asthma phenotype (never asthma, early-onset transient, early-onset persistent and late-onset) was calculated according to parental asthma status (neither parent, maternal only, paternal only or both parents). Crude and adjusted ORs with 95% confidence intervals (CIs) for each phenotype were estimated using logistic regression, with children of non-asthmatic parents as the reference. Adjusted models included maternal age at delivery and parental (maternal and paternal) BMI, education, smoking and atopy.

Because some mothers contributed data for more than one child, observations were not independent. Cluster-robust variance estimation at the maternal level was therefore applied to account for within-family correlation and obtain appropriate standard errors. Additional analyses clustering at the paternal level yielded similar results (online supplemental table 2).

To explore whether associations between parental asthma and offspring asthma phenotypes differed according to allergic predisposition, secondary analyses were stratified by parental atopy. We also conducted a secondary analysis restricted to children with early-onset asthma to examine odds of persistent vs transient disease according to parental asthma status.

To assess the robustness of our findings, we conducted additional sensitivity analyses. First, we analysed a broader sample including children with asthma information from the 3-year questionnaire and either the 7-year or 14-year questionnaire. Second, we repeated analyses restricted to children with explicit yes/no asthma responses at all follow-up ages (3, 7 and 14 years). Third, we applied a stricter definition of persistent asthma requiring asthma at both ages 7 and 14 years, irrespective of asthma status at age 3 years.

All analyses were performed using Stata V. 18.0 (StataCorp, College Station, Texas, USA).

## Results

### Study population

Among 16 055 children, maternal asthma only was reported in 6.6%, paternal asthma only in 8.0% and asthma in both parents in 0.7% (table 1). Baseline characteristics for the study population are presented in table 1. Childhood asthma phenotypes were distributed as follows: never asthma 88.8%, early-onset transient 3.3%, early-onset persistent 2.7% and late-onset 5.2% (online supplemental table 3).

**Table 1:** Baseline characteristics of the study population according to parental asthma status

Characteristics	Parental asthma status			
	Neither parent	Maternal only	Paternal only	Both parents
Children, n (%)	13 590 (84.7%)	1064 (6.6%)	1285 (8.0%)	116 (0.7%)
Sex of child				
Male	6793 (50.0%)	560 (52.6%)	641 (49.9%)	53 (45.7%)
Female	6797 (50.0%)	504 (47.4%)	644 (50.1%)	63 (54.3%)
Birth weight, mean (SD), g	3570 (579.7)	3556 (570.3)	3579 (575.8)	3463 (544.3)
Maternal age at delivery (years)				
<25	798 (5.6%)	81 (7.6%)	100 (7.8%)	16 (13.8%)
25–29	4155 (30.6%)	367 (34.5%)	471 (36.7%)	44 (37.9%)
30–34	5870 (43.2%)	435 (40.9%)	513 (39.9%)	35 (30.2%)
35–39	2436 (17.9%)	158 (14.9%)	180 (14.0%)	18 (15.5%)
40+	341 (2.5%)	23 (2.2%)	21 (1.6%)	3 (2.6%)
Maternal age at delivery, mean (SD), years	31.0 (4.2)	30.5 (4.3)	30.3 (4.2)	29.8 (4.8)
Maternal pre-pregnancy BMI, mean (SD), kg/m²	23.6 (3.7)	24.7 (4.3)	23.6 (3.7)	24.6 (4.9)
Paternal pre-pregnancy BMI, mean (SD), kg/m²	25.7 (3.1)	25.8 (3.2)	26.0 (3.4)	25.8 (3.3)
Maternal atopy				
No	10 235 (75.3%)	442 (41.5%)	966 (75.2%)	45 (38.8%)
Yes	3355 (24.7%)	622 (58.5%)	319 (24.8%)	71 (61.2%)
Paternal atopy				
No	10 707 (78.8%)	824 (77.4%)	618 (48.1%)	54 (46.6%)
Yes	2883 (21.2%)	240 (22.6%)	667 (51.9%)	62 (53.5%)
Maternal education				
9-year elementary school	95 (0.7%)	9 (0.9%)	17 (1.3%)	1 (0.9%)
1-3 years of high school	2818 (20.7%)	245 (23.0%)	275 (21.4%)	35 (30.2%)
4+ years of university/college	10 122 (74.5%)	760 (71.4%)	926 (72.1%)	76 (65.5%)
Missing	*555* (*4.1%*)	*50* (*4.7%*)	*67* (*5.2%*)	*4* (*3.5%*)
Paternal education				
9-year elementary school	336 (2.5%)	31 (2.9%)	34 (2.7%)	1 (0.9%)
1–3 years of high school	4508 (33.2%)	394 (37.0%)	424 (33.0%)	46 (39.7%)
4+ years of university/college	7680 (56.5%)	537 (50.5%)	726 (56.5%)	63 (54.3%)
Missing	*1066* (*7.8%*)	*102* (*9.6%*)	*101* (*7.9%*)	*6* (*5.2%*)
Maternal smoking during pregnancy (up to week 15)				
No	11 835 (87.1%)	915 (86.0%)	1133 (88.2%)	104 (89.7%)
Yes	569 (4.2%)	60 (5.6%)	51 (4.0%)	4 (3.5%)
Missing	*1186* (*8.7%*)	*89* (*8.4%*)	*101* (*7.9%*)	*8* (*6.9%*)
Paternal smoking during pregnancy (up to week 15)				
No	11 521 (84.8%)	874 (82.1%)	1087 (84.6%)	105 (90.5%)
Yes	1917 (14.1%)	180 (16.9%)	185 (14.4%)	11 (9.5%)
*Missing*	*152* (*1.1%*)	*10* (*0.9%*)	*13* (*1.0%*)	*0* (*0.0%*)

Data are n (%) or mean (SD) unless otherwise indicated. Maternal and paternal asthma were self-reported at baseline.

Missing data are shown where applicable.

BMI, body mass index.

#### Associations between parental asthma and offspring asthma phenotypes

Parental asthma was associated with increased odds of all offspring asthma phenotypes, with consistently higher estimates for maternal than paternal asthma, particularly for early-onset persistent asthma (table 2).

**Table 2:** Associations between parental asthma and offspring asthma phenotypes

	Early-onset transient				
Parental asthma status	n	Absolute risk (%)	Risk difference, % (95% CI)	Crude OR (95% CI)	Adjusted OR (95% CI)
Neither parent	386	2.8	0 (Reference)	1 (Reference)	1 (Reference)
Maternal only	66	6.2	3.4 (1.8 to 4.9)	2.26 (1.72 to 2.97)	2.09 (1.52 to 2.88)
Paternal only	67	5.2	2.4 (1.0 to 3.8)	1.88 (1.44 to 2.46)	2.01 (1.48 to 2.73)
Both parents	5	4.3	1.5 (−1.0-4.0)	1.54 (0.62 to 3.80)	0.99 (0.31 to 3.20)

	Early-onset persistent				
Parental asthma status	n	Absolute risk (%)	Risk difference	Crude OR (95% CI)	Adjusted OR (95% CI)
Neither parent	276	2.0	0 (Reference)	1 (Reference)	1 (Reference)
Maternal only	86	8.1	6.1 (4.3–7.9)	4.24 (3.27–5.50)	3.47 (2.56–4.71)
Paternal only	62	4.8	2.8 (1.5–4.1)	2.45 (1.84–3.24)	2.49 (1.81–3.44)
Both parents	13	11.2	9.2 (4.8–13.6)	6.09 (3.37–11.00)	5.34 (2.86–9.98)

	Late-onset				
Parental asthma status	n=	Absolute risk (%)	Risk difference	Crude OR (95% CI)	Adjusted OR (95% CI)
Neither parent	595	4.4	0 (Reference)	1 (Reference)	1 (Reference)
Maternal only	117	11.0	6.6 (5.0–8.2)	2.70 (2.19–3.33)	2.18 (1.69–2.81)
Paternal only	115	9.0	4.6 (3.2–6.0)	2.15 (1.74–2.65)	1.85 (1.44–2.38)
Both parents	13	11.2	6.8 (2.0–11.6)	2.76 (1.54–4.95)	1.96 (0.99–3.86)

Crude and adjusted ORs with 95% confidence intervals (CIs) and absolute risk differences (95% CIs) for early-onset transient, early-onset persistent and late-onset asthma among children, stratified by parental asthma status. The reference group comprised children with neither parent reporting asthma. Adjusted models accounted for maternal age at delivery, maternal and paternal education, maternal and paternal pre-pregnancy BMI, maternal and paternal smoking during pregnancy and maternal and paternal atopy. Cluster-robust variance estimation at the maternal level accounted for sibling correlation.

BMI, body mass index.

For early-onset transient asthma, both maternal and paternal asthma were associated with increased odds compared with children of non-asthmatic parents, with adjusted ORs of 2.09 (95% CI 1.52 to 2.88) for maternal-only asthma and 2.01 (95% CI 1.48 to 2.73) for paternal-only asthma. The number of early-onset transient cases among children with asthma in both parents was small, limiting interpretation (adjusted OR 0.99, 95% CI 0.31 to 3.20).

The strongest associations were observed for early-onset persistent asthma. Maternal asthma was associated with an adjusted OR 3.47, 95% CI 2.56 to 4.71 and paternal asthma with an adjusted OR 2.49, 95% CI 1.81 to 3.44. Children with asthma in both parents had the highest odds (adjusted OR 5.34, 95% CI 2.86 to 9.98).

For late-onset asthma, both maternal and paternal asthma were associated with increased odds (maternal asthma: adjusted OR 2.18, 95% CI 1.69 to 2.81; paternal asthma: adjusted OR 1.85, 95% CI 1.44 to 2.38). Asthma in both parents was also associated with increased odds, although the CI included the null (adjusted OR 1.96, 95% CI 0.99 to 3.86).

#### Early-onset asthma: persistent versus transient disease

Among children with early-onset asthma (n=961), maternal asthma was associated with higher odds of persistent versus transient disease (OR 1.82, 95% CI 1.27 to 2.61), whereas paternal asthma showed a weaker association (OR 1.29, 95% CI 0.89 to 1.89) (online supplemental table 4).

#### Sex-specific patterns

Parental asthma showed some variation by offspring sex (online supplemental table 5). For early-onset transient asthma, maternal-only asthma was associated with similar odds in males (OR 2.01, 95% CI 1.35 to 3.01) and females (OR 2.12, 95% CI 1.24 to 3.62). Effect estimates for paternal-only asthma were larger in males (OR 2.60, 95% CI 1.81 to 3.74) than females (OR 1.12, 95% CI 0.60 to 2.07), although CIs overlapped. For early-onset persistent and late-onset asthma, maternal and paternal associations were generally comparable across sexes (online supplemental table 5).

#### Parental atopy and offspring asthma

Associations similar across strata defined by maternal and paternal atopy (tables 3 and 4). For early-onset transient asthma, associations with maternal and paternal asthma were modest and comparable regardless of parental atopy status.

**Table 3:** Associations between parental asthma and offspring asthma phenotypes, stratified by maternal atopy

	Offspring asthma phenotype							
Parental asthma status	Early-onset transient asthma							
	n				Crude OR (95% CI)		Adjusted OR (95% CI)	
	Maternal atopy		No Maternal atopy		Maternal atopy	No Maternal atopy	Maternal atopy	No Maternal atopy
	Early-Onset transient asthma	No asthma	Early-Onset transient asthma	No asthma				
Neither parent	126	3229	260	9975	1 (Reference)	1 (Reference)	1 (Reference)	1 (Reference)
Maternal only	35	587	31	411	1.53 (1.04 to 2.24)	2.89 (1.95–4.30)	1.59 (1.03 to 2.44)	2.86 (1.84–4.44)
Paternal only	19	300	48	918	1.62 (0.96 to 2.73)	2.00 (1.46–2.75)	1.71 (0.98 to 2.97)	2.04 (1.42–2.92)
Both parents	2	69	3	42	0.74 (0.17 to 3.07)	2.74 (0.84–8.92)	0.87 (0.21 to 3.62)	1.00 (0.14–7.36)

Parental asthma status	Early-onset persistent asthma							
	n				Crude OR (95% CI)		Adjusted OR (95% CI)	
	Maternal atopy		No Maternal atopy		Maternal atopy	No Maternal atopy	Maternal atopy	No Maternal atopy
	Early-onset persistent asthma	No asthma	Early-onset persistent asthma	No asthma				
Neither parent	86	3269	190	10 045	1 (Reference)	1 (Reference)	1 (Reference)	1 (Reference)
Maternal only	63	559	23	419	4.28 (3.01 to 6.09)	2.90 (1.86–4.52)	3.49 (2.32 to 5.25)	3.64 (2.28–5.83)
Paternal only	24	295	38	928	3.09 (1.94 to 4.94)	2.16 (1.52–3.09)	2.90 (1.74 to 4.85)	2.31 (1.56–3.42)
Both parents	10	61	3	42	6.23 (3.08 to 12.62)	3.78 (1.16–12.32)	5.71 (2.66 to 12.26)	4.41 (1.39–14.00)

Parental asthma status	Late-onset asthma							
	n				Crude OR (95% CI)		Adjusted OR (95% CI)	
	Maternal atopy		No Maternal atopy		Maternal atopy	No Maternal atopy	Maternal atopy	No Maternal atopy
	Late-onset asthma	No asthma	Late-onset asthma	No asthma				
Neither parent	176	3179	419	9816	1 (Reference)	1 (Reference)	1 (Reference)	1 (Reference)
Maternal only	82	540	35	407	4.28 (3.01 to 6.09)	2.90 (1.86–4.52)	2.53 (1.82 to 3.52)	1.70 (1.09–2.64)
Paternal only	37	282	78	888	3.09 (1.94 to 4.94)	2.16 (1.52–3.09)	2.11 (1.34 to 3.31)	2.07 (1.54–2.79)
Both parents	8	63	5	40	6.23 (3.07 to 12.62)	3.78 (1.16–12.32)	1.90 (0.79 to 4.54)	2.83 (0.99–8.14)

Crude and adjusted ORs with 95% CIs for associations between parental asthma status and offspring asthma phenotypes, stratified by maternal atopy (yes/no). Adjustment included maternal age at delivery, maternal and paternal education, maternal and paternal pre-pregnancy BMI and maternal and paternal smoking during pregnancy. Cluster-robust variance estimation at the maternal level was used to account for correlation between siblings. Reference group=children with neither parent reporting asthma.

BMI, body mass index.

**Table 4:** Associations between parental asthma and offspring asthma phenotypes, stratified by paternal atopy

	Offspring asthma phenotype							
Parental asthma status	Early-onset transient asthma							
	n				Crude OR (95% CI)		Adjusted OR (95% CI)	
	Paternal atopy		No Paternal atopy		Paternal atopy	No Paternal atopy	Paternal atopy	No Paternal atopy
	Early-onset asthma	No asthma	Early-onset asthma	No asthma				
Neither parent	73	2810	313	10 394	1 (Reference)	1 (Reference)	1 (Reference)	1 (Reference)
Maternal only	16	224	50	774	2.75 (1.57 to 4.80)	2.15 (1.57–2.93)	2.44 (1.31 to 4.56)	2.25 (1.59–3.19)
Paternal only	33	634	34	584	2.00 (1.31 to 3.07)	1.93 (1.34–2.78)	2.11 (1.32 to 3.37)	1.94 (1.28–2.94)
Both parents	2	60	3	51	1.28 (0.31 to 5.36)	1.95 (0.61–6.30)	0.67 (0.09 to 4.84)	1.55 (0.37–6.52)

Parental asthma status	Early-onset persistent asthma							
	n				Crude OR (95% CI)		Adjusted OR (95% CI)	
	Paternal atopy		No Paternal atopy		Paternal atopy	No Paternal atopy	Paternal atopy	No Paternal atopy
	Early-onset persistent asthma	No asthma	Early-onset persistent asthma	No asthma				
Neither parent	62	2821	214	10 493	1 (Reference)	1 (Reference)	1 (Reference)	1 (Reference)
Maternal only	27	213	59	765	5.77 (3.47 to 9.57)	3.78 (2.80–5.11)	5.54 (3.14 to 9.77)	3.64 (2.58–5.14)
Paternal only	30	637	32	586	2.14 (1.37 to 3.34)	2.68 (1.83–3.92)	2.23 (1.36 to 3.65)	2.77 (1.82–4.21)
Both parents	6	56	7	47	4.88 (2.02 to 11.78)	7.30 (3.25–16.39)	4.56 (1.73 to 12.01)	8.67 (3.86–19.47)

Parental asthma status	Late-onset asthma							
	n				Crude OR (95% CI)		Adjusted OR (95% CI)	
	Paternal atopy		No Paternal atopy		Paternal atopy	No Paternal atopy	Paternal atopy	No Paternal atopy
	Late-onset asthma	No asthma	Late-onset asthma	No asthma				
Neither parent	152	2731	443	10 264	1 (Reference)	1 (Reference)	1 (Reference)	1 (Reference)
Maternal only	37	203	80	744	3.27 (2.21 to 4.86)	2.49 (1.94–3.20)	2.80 (1.78 to 4.42)	2.32 (1.72–3.13)
Paternal only	68	599	47	571	2.04 (1.50 to 2.77)	1.91 (1.40–2.60)	2.07 (1.46 to 2.93)	1.68 (1.15–2.45)
Both parents	8	54	5	49	2.66 (1.24 to 5.72)	2.36 (0.94–5.97)	2.35 (0.99 to 5.60)	2.33 (0.81–6.69)

Crude and adjusted ORs with 95% CIs for associations between parental asthma status and offspring asthma phenotypes, stratified by paternal atopy (yes/no). Adjustment included maternal age at delivery, maternal and paternal education, maternal and paternal pre-pregnancy BMI, and maternal and paternal smoking during pregnancy. Cluster-robust variance estimation at the maternal level was used to account for correlation between siblings. Reference group=children with neither parent reporting asthma.

BMI, body mass index.

For early-onset persistent asthma, maternal asthma showed stronger associations than paternal asthma across atopy strata, and children with asthma in both parents had the highest odds. Associations with late-onset asthma were generally weaker than those observed for early-onset persistent asthma.

#### Sensitivity analyses

In a sensitivity analysis including children who completed the 3-year questionnaire and either the 7-year or 14-year questionnaire (online supplemental table 6), results were consistent with the primary analysis. Estimates were similar in direction across parental exposure groups, with slightly higher ORs in smaller subgroups, particularly for children with asthma in both parents (eg, early-onset persistent: adjusted OR 6.28, 95% CI 4.16 to 9.48).

Restriction to children with explicit yes/no responses to asthma questions produced similar results, although CIs were wider due to reduced sample size (online supplemental table 7). Using an alternative definition of early-onset persistent asthma requiring asthma at both 7 and 14 years likewise showed similar associations (online supplemental table 8), although with less precision due to fewer cases.

#### Additional analysis

Excluding parental atopy from the models resulted in slightly stronger but similar associations (online supplemental table 9).

Across analyses, estimates for smaller subgroups, particularly children with asthma in both parents, were imprecise because of limited numbers.

## Discussion

### Phenotype-specific findings

In this population-based cohort of over 16 000 children, parental asthma was associated with increased odds of offspring asthma across all phenotypes, with higher point estimates for maternal than for paternal asthma. However, CIs overlapped for most phenotypes, and maternal–paternal differences should be interpreted cautiously. The strongest associations were observed for early-onset persistent asthma, particularly when both parents were affected. Associations with paternal asthma were generally smaller, especially for late-onset asthma.

Early-onset transient asthma showed similar associations for maternal and paternal asthma, in contrast to the more pronounced maternal predominance observed for early-onset persistent disease, possibly reflecting underlying phenotypic heterogeneity. Early-onset transient asthma is often considered a heterogeneous wheezing phenotype with weaker familial and atopic contributions and greater susceptibility to early-life environmental exposures, including viral infections, tobacco smoke and air pollution.^22–24^ Although these exposures are common, only a subset of children develop recurrent wheeze or asthma, suggesting heterogeneity in individual susceptibility.^25^

While environmental exposures are also important in early-onset persistent asthma, this phenotype has been shown to exhibit stronger familial aggregation and closer associations with atopy and allergic disease, as demonstrated in previous birth cohorts including ALSPAC and other population-based studies.^9 22 26 27^ The stronger associations observed for early-onset persistent asthma may be consistent with a phenotype characterised by a greater contribution of allergic sensitisation and familial susceptibility than transient early wheezing phenotypes.^9 14^

### Maternal versus paternal effects

Our findings are consistent with previous studies suggesting stronger associations with maternal than paternal asthma and offspring disease. A meta-analysis by Lim *et al* found that maternal asthma was associated with a greater increase in offspring asthma risk than paternal asthma, suggesting that maternal effects may extend beyond shared genetic inheritance.^5^ Similarly, Liu *et al* found that poorer asthma control during pregnancy was associated with increased risk of offspring asthma, particularly early-onset persistent asthma.^13^

However, although point estimates for maternal asthma were generally higher than for paternal asthma, CIs overlapped for most phenotypes, limiting evidence for clear parent-specific effects. This cautious interpretation is supported by findings from the Danish General Suburban Population Study (GESUS), which reported similar maternal and paternal associations with offspring asthma.^28^ Differences in cohort definitions and reporting across studies may contribute to variation in maternal–paternal contrasts.

In this cohort, paternal asthma was slightly more prevalent than maternal asthma, which contrasts with reports of higher asthma prevalence among adult women than men in many populations.^29 30^ The reasons for this difference are unclear and may be cohort-specific.

### Mechanistic considerations

Several mechanisms may contribute to the stronger associations observed with maternal asthma than paternal asthma. This pattern may be consistent with a phenotype in which familial and atopic mechanisms play a greater role. Maternal-specific influences, including in utero immune modulation, epigenetic programming during foetal development and the maternal microbiome, have been implicated in childhood asthma risk.^13 31 32^

Maternal associations persisted after stratification by parental atopy (tables 3 and 4), suggesting that effects were not fully explained by shared allergic predisposition. Excluding parental atopy from the adjusted models had little influence on the effect estimates (online supplemental table 9). Although shared prenatal mechanisms have been proposed across childhood asthma and other allergic diseases,^33^ our findings did not suggest additional associations related to parental allergy-related diseases beyond those associated with parental asthma. Together with weaker paternal associations, these findings are consistent with prenatal and early-life non-genetic mechanisms.

The stronger maternal association for early-onset persistent asthma, compared with transient asthma, possibly reflects a phenotype characterised by greater familial susceptibility and atopic contribution. In contrast, parental atopy appeared to play a limited role in early-onset transient asthma, suggestive of weaker familial and allergic associations and greater sensitivity to early-life environmental exposures.^22–24^ Associations with late-onset asthma were generally smaller than those observed for early-onset persistent asthma. These stratified findings should be interpreted cautiously due to limited precision, particularly in smaller subgroups.

Sex-stratified analyses suggested some variation, but overlapping CIs indicate limited evidence for clear sex differences. These patterns may reflect differences in lung development, hormonal influences or immune responses between boys and girls.^30 34 35^ While differences in asthma risk by sex are well documented—for example, higher rates of early-life wheezing and asthma in boys—the influence of parental asthma on offspring asthma phenotypes by sex remains less well characterised.^35 36^ The strong associations among children with asthma in both parents may reflect additive familial susceptibility and shared environmental influences, although estimates were based on small numbers.^5 37^

Overall, these results, together with our previous findings demonstrating persistent maternal effects across childhood and adolescence,^16^ suggest that maternal asthma is particularly relevant for offspring phenotypes characterised by early onset and persistence. Clinically, these findings highlight the importance of detailed family history, particularly maternal asthma, in assessing asthma risk. These mechanistic interpretations remain speculative and should be considered hypothesis-generating given the observational design of the study.

### Strengths and limitations

Key strengths of this study include the large, population-based design with longitudinal follow-up from early childhood through adolescence, enabling robust classification of distinct asthma phenotypes based on age at onset and persistence. Repeated assessments at ages 3, 7 and 14 years minimised outcome misclassification, while prospective reporting of parental asthma reduced recall bias. Inclusion of both maternal and paternal asthma allowed direct parent-specific comparisons, while sex-stratified and parental atopy–stratified analyses provided additional insights into potential effect modification. Observed maternal asthma effects, particularly for early-onset persistent asthma, persisted across strata of parental atopy, suggesting that maternal associations were not fully explained by parental atopy.

Several limitations should be considered. First, parental asthma was based on self-reported history of asthma, and independent validation against clinical records or registry data was not available. Differences in question wording between mothers and fathers may have introduced some heterogeneity in exposure classification. However, both parental exposures were assessed prospectively at recruitment and referred to a history of asthma before or up to the time of enrolment. Any resulting misclassification is likely non-differential with respect to offspring asthma phenotype and would therefore be expected to bias associations towards the null rather than fully explain the consistently higher maternal point estimates observed across phenotypes.

Second, childhood asthma was based on parental report and was not clinically verified. Maternal reporting may be influenced by awareness or care-seeking behaviours, with asthmatic mothers potentially more likely to notice or report symptoms in their children, contributing to stronger maternal-offspring associations. However, such awareness or reporting bias may also apply to fathers with asthma.

Maternally reported asthma in MoBa has previously been validated against the Norwegian Prescription Database, showing high specificity (96.8%) and good sensitivity (approximately 80–85%) for asthma and antiasthmatic medication use.^21^ These findings suggest that maternally reported asthma is a reasonable proxy for clinically relevant asthma in large population-based studies.

Third, potential selection bias should be considered. The initial MoBa cohort participation rate was 41%, and participation is known to be socially patterned, with lower response among individuals with lower education and more adverse health behaviours, including smoking.^38 39^ Such selective participation may affect prevalence estimates and may introduce bias if both exposure and outcome are associated with participation.

Attrition from the full cohort to the analytic sample of 16 055 children reflected broadly similar sociodemographic patterns for mothers and fathers (figure 1 and online supplemental table 1). Maternal asthma prevalence remained stable across follow-up (7.6% in the full MoBa cohort vs 7.4% at age 14), suggesting no strong evidence of differential loss by exposure status. Missing data were primarily due to questionnaire non-response rather than incomplete covariate information.

Although we did not apply inverse probability weighting or formal quantitative bias analyses to account for potential selection bias,^40 41^ we compared baseline characteristics between included and excluded participants to improve transparency regarding attrition patterns (online supplemental table 1). Previous methodological studies in MoBa indicate that exposure–outcome associations are generally robust despite selective participation and attrition.^38 39^ Although residual selection bias cannot be excluded, broadly similar attrition patterns across parental exposure groups reduce concerns regarding substantial bias due to differential selection.

Additional limitations include possible overadjustment if parental atopy lies partly on the causal pathway between parental and offspring asthma phenotypes. However, additional analyses excluding adjustment for parental atopy yielded similar overall patterns of association with only modest differences in effect estimates. Finally, multiple testing and exploratory subgroup analyses increase the possibility of type I error. Some subgroup analyses, particularly those involving asthma in both parents, were based on small numbers and should therefore be interpreted cautiously.

### Implications

Distinguishing asthma phenotypes and considering both parental and child sex may help characterise familial risk patterns. Maternal asthma showed the most consistent associations, suggesting a potential role for prenatal and early-life influences in offspring asthma risk. Recognition of these phenotype-specific patterns may improve identification of children at higher familial risk in clinical and research settings.

## Conclusion

Maternal asthma was more consistently associated with offspring asthma phenotypes than paternal asthma, particularly for early-onset persistent asthma, although CIs overlapped for most comparisons. Children with asthma in both parents had the highest odds of asthma, suggesting additive familial susceptibility and shared environmental influences, but estimates were imprecise due to small numbers.

Early-onset transient asthma showed weaker associations with parental asthma and atopy, consistent with a more heterogeneous phenotype influenced by early-life environmental exposures and individual susceptibility. Maternal associations were observed across parental atopy strata, supporting effects beyond allergic predisposition.

Overall, these findings highlight heterogeneity in childhood asthma phenotypes and the importance of considering parental asthma when interpreting familial risk. Further studies are needed to clarify mechanisms underlying potential maternal influences on persistent asthma.

## Supplementary material

10.1136/bmjresp-2026-004245 online supplemental table 1

10.1136/bmjresp-2026-004245 online supplemental table 2

10.1136/bmjresp-2026-004245 online supplemental table 3

10.1136/bmjresp-2026-004245 online supplemental table 4

10.1136/bmjresp-2026-004245 online supplemental table 5

10.1136/bmjresp-2026-004245 online supplemental table 6

10.1136/bmjresp-2026-004245 online supplemental table 7

10.1136/bmjresp-2026-004245 online supplemental table 8

10.1136/bmjresp-2026-004245 online supplemental table 9

10.1136/bmjresp-2026-004245 online supplemental figure 1

10.1136/bmjresp-2026-004245 online supplemental file 1

## Data Availability

Data are available upon reasonable request. Data may be obtained from a third party and are not publicly available.
