# Supplementary material for: Parental asthma and childhood asthma phenotypes: maternal associations with persistent asthma
Source: BMJ Open Respir Res. 2026 Aug 4;13(1):e004245. doi: 10.1136/bmjresp-2026-004245 (PMC13448598; doi:10.1136/bmjresp-2026-004245)
Supplement: Supplementary file 2 [file bmjresp-13-1-s002.docx]

**Supplementary table 2:** **Crude and adjusted odds ratios (ORs) with 95% confidence intervals (CIs) for childhood asthma phenotypes according to parental asthma status using cluster-robust variance estimation at the paternal level.**

|  | **Early-onset transient** | | |
| --- | --- | --- | --- |
| **Parental asthma status** | **Crude OR**  **(95% CI)** | | **Adjusted OR (95% CI)** |
| **Neither parent** | 1 (Reference) | | 1 (Reference) |
| **Maternal only** | 2.26  (1.72-2.97) | | 2.09  (1.52-2.88) |
| **Paternal only** | 1.88  (1.44-2.46) | | 2.01  (1.48-2.74) |
| **Both parents** | 1.54  (0.62-3.80) | | 0.99  (0.31-3.20) |
|  | **Early-onset persistent** | | |
| **Parental asthma status** | **Crude OR (95% CI)** | **Adjusted OR (95% CI)** | |
| **Neither parent** | 1 (Reference) | 1 (Reference) | |
| **Maternal only** | 4.24  (3.27-5.50) | 3.47  (2.45-4.71) | |
| **Paternal only** | 2.45  (1.85-3.25) | 2.50  (1.81-3.45) | |
| **Both parents** | 6.08  (3.37-11.00) | 5.34  (2.86-9.98) | |
|  | **Late-onset** | | |
| **Parental asthma status** | **Crude OR (95% CI)** | **Adjusted OR (95% CI)** | |
| **Neither parent** | 1 (Reference) | 1 (Reference) | |
| **Maternal only** | 2.70  (2.19-3.33) | 2.18  (1.69-2.81) | |
| **Paternal only** | 2.15  (1.74-2.65) | 1.85  (1.44-2.37) | |
| **Both parents** | 2.76  (1.54-4.95) | 1.96  (0.99-3.86) | |

The reference group comprised children with neither parent reporting asthma. Adjusted models included maternal age at delivery, maternal and paternal education, maternal and paternal pre-pregnancy BMI, maternal and paternal smoking during pregnancy, and maternal and paternal atopy. Cluster-robust variance estimation at the paternal level was used to assess robustness to alternative clustering specification. BMI, body mass index.
