# Supplementary material for: Parental asthma and childhood asthma phenotypes: maternal associations with persistent asthma
Source: BMJ Open Respir Res. 2026 Aug 4;13(1):e004245. doi: 10.1136/bmjresp-2026-004245 (PMC13448598; doi:10.1136/bmjresp-2026-004245)
Supplement: Supplementary file 3 [file bmjresp-13-1-s003.docx]

**Supplementary Table 3. Distribution of asthma phenotypes in a cohort of 16,055 children.**

| **Asthma phenotype** | **Children n (%)** |
| --- | --- |
| **Never** | 14254 (88.8%) |
| **Early-onset transient** | 524 (3.3%) |
| **Early-onset persistent** | 437 (2.7%) |
| **Late-onset** | 840 (5.2%) |

Asthma phenotypes were classified as never, early-onset transient, early-onset persistent, or late-onset. Values are presented as number of children (percentage).
