# Supplementary material for: Parental asthma and childhood asthma phenotypes: maternal associations with persistent asthma
Source: BMJ Open Respir Res. 2026 Aug 4;13(1):e004245. doi: 10.1136/bmjresp-2026-004245 (PMC13448598; doi:10.1136/bmjresp-2026-004245)
Supplement: Supplementary file 4 [file bmjresp-13-1-s004.docx]

|  | **Early-onset persistent asthma (vs transient)** | |
| --- | --- | --- |
| **Parental asthma status** | **Crude OR**  **(95% CI)** | **Adjusted OR (95% CI)** |
| **Neither parent** | 1 (Reference) | 1 (Reference) |
| **Maternal only** | 1.82  (1.27-2.62) | 1.73  (1.14-2.63) |
| **Paternal only** | 1.29  (0.89-1.89) | 1.26  (0.81-1.95) |
| **Both parents** | 3.64  (1.28-10.33) | 4.67  (1.21-18.11) |

**Supplemental table 4.** **Crude and adjusted odds ratios (ORs) with 95% confidence intervals (95% CIs) for persistent (vs transient) asthma, among children with early-onset asthma.**

Adjusted models accounted for maternal age at delivery, maternal and paternal education, maternal and paternal pre-pregnancy BMI, maternal and paternal smoking during pregnancy, and maternal and paternal atopy. Cluster-robust variance estimation at the maternal level accounted for sibling correlation. BMI, Body Mass Index.
