# Supplementary material for: Parental asthma and childhood asthma phenotypes: maternal associations with persistent asthma
Source: BMJ Open Respir Res. 2026 Aug 4;13(1):e004245. doi: 10.1136/bmjresp-2026-004245 (PMC13448598; doi:10.1136/bmjresp-2026-004245)
Supplement: Supplementary file 5 [file bmjresp-13-1-s005.docx]

**Supplementary Table 5. Sex-stratified adjusted odds ratios for offspring asthma phenotypes by parental asthma status.**

| **Parental asthma status** | **Early-onset transient** | | **Early-onset persistent** | | **Late onset** | |
| --- | --- | --- | --- | --- | --- | --- |
|  | ***Male*** | ***Female*** | ***Male*** | ***Female*** | ***Male*** | ***Female*** |
| **Neither parent** | 1 | 1 | 1 | 1 | 1 | 1 |
| **Maternal only** | 2.01 (1.35-3.01) | 2.12 (1.24-3.62) | 4.04 (2.79-5.86) | 2.53 (1.50-4.27) | 1.91 (1.34-2.73) | 2.48 (1.73-3.57) |
| **Paternal only** | 2.60 (1.81-3.74) | 1.12 (0.60-2.07) | 2.81 (1.88-4.20) | 2.09 (1.22-3.57) | 1.57 (1.10-2.23) | 2.21 (1.55-3.15) |
| **Both parents** | 0.59 (0.08-4.51) | 1.66 (0.40-6.95) | 5.82 (2.41-14.06) | 4.98 (2.03-12.20) | 1.66 (0.58-4.72) | 2.22 (0.89-5.52) |

Adjusted odds ratios (aORs) with 95% confidence intervals (CIs) for early-onset transient, early-onset persistent, and late-onset asthma, calculated separately for male and female offspring. The reference group comprises children with neither parent reporting asthma. Models were adjusted for maternal age at delivery, maternal and paternal education, maternal and paternal pre-pregnancy BMI, maternal and paternal smoking during pregnancy, and maternal and paternal atopy. Cluster-robust variance estimation at the maternal level accounted for correlation between siblings. BMI, body mass index.
