# Supplementary material for: Parental asthma and childhood asthma phenotypes: maternal associations with persistent asthma
Source: BMJ Open Respir Res. 2026 Aug 4;13(1):e004245. doi: 10.1136/bmjresp-2026-004245 (PMC13448598; doi:10.1136/bmjresp-2026-004245)
Supplement: Supplementary file 6 [file bmjresp-13-1-s006.docx]

**Supplementary Table 6. Sensitivity analysis: Associations between parental asthma and offspring asthma phenotypes (expanded sample).**

|  | **Early-onset transient** | | | | | | | |
| --- | --- | --- | --- | --- | --- | --- | --- | --- |
| **Parental asthma status** | **n** | **Absolute risk (%)** | | **Risk difference, % (95% CI)** | | **Crude OR**  **(95% CI)** | | **Adjusted OR (95% CI)** |
| **Neither parent** | 957 | 3.1 | | 0 (Reference) | | 1 (Reference) | | 1 (Reference) |
| **Maternal only** | 176 | 7.4 | | 4.3 (3.2-5.4) | | 2.50 (2.12-2.96) | | 2.42  (1.99-2.95) |
| **Paternal only** | 165 | 5.6 | | 2.5 (1.7-3.4) | | 1.87 (1.58-2.21) | | 1.95  (1.61-3.37) |
| **Both parents** | 25 | 9.9 | | 6.8 (3.1-10.5) | | 3.44 (2.23-5.30) | | 2.05  (1.15-3.64) |
|  | **Early-onset persistent** | | | | | | | |
| **Parental asthma status** | **n** | | **Absolute risk** | **Risk difference** | **Crude OR (95% CI)** | | **Adjusted OR (95% CI)** | |
| **Neither parent** | 658 | | 2.1 | 0 (Reference) | 1 (Reference) | | 1 (Reference) | |
| **Maternal only** | 186 | | 7.8 | 5.7 (4.6-6.8) | 3.90  (3.28-4.66) | | 3.08  (2.49-3.81) | |
| **Paternal only** | 147 | | 5.0 | 2.9 (2.1-3.7) | 2.43  (2.02-2.92) | | 2.43  (1.96-3.01) | |
| **Both parents** | 36 | | 14.2 | 12.1 (7.8-16.4) | 7.64  (5.33-10.97) | | 6.28  (4.16-9.48) | |
|  | **Late-onset** | | | | | | | |
| **Parental asthma status** | **n** | | **Absolute risk** | **Risk difference** | **Crude OR (95% CI)** | | **Adjusted OR (95% CI)** | |
| **Neither parent** | 976 | | 3.2 | 0 (Reference) | 1 (Reference) | | 1 (Reference) | |
| **Maternal only** | 210 | | 8.8 | 5.6 (4.5-6.8) | 2.94  (2.54-3.48) | | 2.52  (2.09-3.03) | |
| **Paternal only** | 182 | | 6.2 | 3.0 (2.2-3.9) | 2.03  (1.72-2.39) | | 1.76  (1.44-2.15) | |
| **Both parents** | 24 | | 9.5 | 6.3 (2.7-10.0) | 3.22  (2.12-4.90) | | 2.51  (1.53-4.12) | |

Sensitivity analysis including children with the 3-year questionnaire and either 7- or 14-year follow-up. Total number of participants by this method was 36547. Adjusted odds ratios (ORs) with 95% confidence intervals (CIs) for early-onset transient, early-onset persistent, and late-onset asthma are shown. Adjusted models accounted for maternal age at delivery, maternal and paternal education, maternal and paternal pre-pregnancy BMI, maternal and paternal smoking during pregnancy and maternal and paternal atopy. Cluster-robust variance estimation at the maternal level accounted for sibling correlation. BMI, Body Mass Index.
