# Supplementary material for: Parental asthma and childhood asthma phenotypes: maternal associations with persistent asthma
Source: BMJ Open Respir Res. 2026 Aug 4;13(1):e004245. doi: 10.1136/bmjresp-2026-004245 (PMC13448598; doi:10.1136/bmjresp-2026-004245)
Supplement: Supplementary file 7 [file bmjresp-13-1-s007.docx]

|  | **Early-onset transient** | |
| --- | --- | --- |
| **Parental asthma status** | **Crude OR**  **(95% CI)** | **Adjusted OR (95% CI)** |
| **Neither parent** | 1 (Reference) | 1 (Reference) |
| **Maternal only** | 2.29 (1.51-3.47) | 2.14 (1.32-3.47) |
| **Paternal only** | 1.84 (1.21-2.80) | 1.83 (1.13-2.99) |
| **Both parents** | 0.80 (0.11-5.76) | Not estimable* |
|  | **Early-onset persistent** | |
| **Parental asthma status** | **Crude OR (95% CI)** | **Adjusted OR (95% CI)** |
| **Neither parent** | 1 (Reference) | 1 (Reference) |
| **Maternal only** | 3.40 (2.72-4.26) | 2.97 (2.28-3.86) |
| **Paternal only** | 2.25 (1.78-2.86) | 2.37 (1.81-3.10) |
| **Both parents** | 4.17 (2.35-7.40) | 3.92 (2.16-7.14) |
|  | **Late-onset** | |
| **Parental asthma status** | **Crude OR (95% CI)** | **Adjusted OR (95% CI)** |
| **Neither parent** | 1 (Reference) | 1 (Reference) |
| **Maternal only** | 2.38 (1.98-2.86) | 1.93 (1.55-2.41) |
| **Paternal only** | 1.92 (1.60-2.31) | 1.60 (1.29-1.99) |
| **Both parents** | 2.62 (1.57-4.35) | 1.72 (0.93-3.19) |

**Supplementary Table 7.** **Sensitivity analysis restricted to children with complete asthma information (yes/no responses) at ages 3, 7, and 14 years, excluding those with missing asthma data at any time point. Crude and adjusted odds ratios (ORs) with 95% confidence intervals (CIs) are shown.**

Adjusted models accounted for maternal age at delivery, maternal and paternal education, pre-pregnancy BMI, smoking during pregnancy, and atopic status in both parents. Cluster-robust variance estimation at the maternal level was used to account for sibling correlation. BMI, body mass index.

* Adjusted estimates for the “both parents with asthma” category in early-onset transient asthma could not be estimated due to sparse data leading to model non-convergence (complete separation).
