# Supplementary material for: Parental asthma and childhood asthma phenotypes: maternal associations with persistent asthma
Source: BMJ Open Respir Res. 2026 Aug 4;13(1):e004245. doi: 10.1136/bmjresp-2026-004245 (PMC13448598; doi:10.1136/bmjresp-2026-004245)
Supplement: Supplementary file 8 [file bmjresp-13-1-s008.docx]

|  | **Offspring persistent asthma** | |
| --- | --- | --- |
| **Parental asthma status** | **Crude OR (95% CI)** | **Adjusted OR (95% CI)** |
| **Neither parent** | 1 (Reference) | 1 (Reference) |
| **Maternal only** | 4.99 (3.24–7.68) | 4.49 (2.66–7.55) |
| **Paternal only** | 3.59 (2.35–5.49) | 3.96 (2.47–6.35) |
| **Both parents** | 11.72 (5.53–24.83) | 12.40 (5.61–27.41) |
|  |  |  |

**Supplementary table 8: Sensitivity analysis presenting crude and adjusted odds ratios (ORs) with 95% confidence intervals (CIs) for offspring persistent asthma, using a stricter definition of persistent asthma (asthma present at both ages 7 and 14 years, irrespective of asthma status at age 3 years).**

Adjusted models accounted for maternal age at delivery, maternal and paternal education, maternal and paternal pre-pregnancy BMI, maternal and paternal smoking during pregnancy, and maternal and paternal atopy. Cluster-robust variance estimation at the maternal level accounted for sibling correlation. BMI, Body Mass Index.
