# Supplementary material for: Parental asthma and childhood asthma phenotypes: maternal associations with persistent asthma
Source: BMJ Open Respir Res. 2026 Aug 4;13(1):e004245. doi: 10.1136/bmjresp-2026-004245 (PMC13448598; doi:10.1136/bmjresp-2026-004245)
Supplement: Supplementary file 9 [file bmjresp-13-1-s009.docx]

|  | **Early-onset transient** | | |
| --- | --- | --- | --- |
| **Parental asthma status** | **Adjusted OR (95% CI)**  **With adjustment for parental atopy** | | **Adjusted OR (95% CI)**  **Without adjustment for parental atopy** |
| **Neither parent** | 1 (Reference) | | 1 (Reference) |
| **Maternal only** | 2.09  (1.52-2.88) | | 2.29  (1.69-3.10) |
| **Paternal only** | 2.01  (1.48-2.74) | | 1.95  (1.44-2.63) |
| **Both parents** | 0.99  (0.31-3.20) | | 1.06  (0.33-3.38) |
|  | **Early-onset persistent** | | |
| **Parental asthma status** | **Adjusted OR (95% CI)**  **With adjustment for parental atopy** | **Adjusted OR (95% CI)**  **Without adjustment for parental atopy** | |
| **Neither parent** | 1 (Reference) | 1 (Reference) | |
| **Maternal only** | 3.47  (2.56-4.71) | 4.08  (3.04-5.48) | |
| **Paternal only** | 2.49  (1.81-3.44) | 2.53  (1.85-3.45) | |
| **Both parents** | 5.34  (2.86-9.98) | 6.41  (3.48-11.82) | |
|  | **Late-onset** | | |
| **Parental asthma status** | **Adjusted OR (95% CI)**  **With adjustment for parental atopy** | **Adjusted OR (95% CI)**  **Without adjustment for parental atopy** | |
| **Neither parent** | 1 (Reference) | 1 (Reference) | |
| **Maternal only** | 2.18  (1.69-2.81) | 2.47  (1.92-3.17) | |
| **Paternal only** | 1.85  (1.44-2.38) | 2.09  (1.63-2.67) | |
| **Both parents** | 1.96  (0.99-3.86) | 2.53  (1.30-4.92) | |

**Supplementary Table 9. Adjusted odds ratios (ORs) with 95% confidence intervals (CIs) for associations between parental asthma status and childhood asthma phenotypes, with and without adjustment for parental atopy.**

Adjusted models included maternal age at delivery, maternal and paternal education, maternal and paternal pre-pregnancy BMI, and maternal and paternal smoking during pregnancy. Models labelled “with adjustment for parental atopy” additionally included maternal and paternal atopy. Cluster-robust variance estimation at the maternal level was used to account for sibling correlation. BMI, body mass index.
