# Supplementary material for: Parental asthma and childhood asthma phenotypes: maternal associations with persistent asthma
Source: BMJ Open Respir Res. 2026 Aug 4;13(1):e004245. doi: 10.1136/bmjresp-2026-004245 (PMC13448598; doi:10.1136/bmjresp-2026-004245)
Supplement: Supplementary file 10 [file bmjresp-13-1-s011.docx]

Supplemental Figure 1: Diagram of confounders. Covariates having the potential to influence both the risk of parental asthma as well as offspring asthma were considered confounders and adjusted for. BMI, body mass index. Parental atopy was included as a covariate reflecting shared familial predisposition to allergic disease. However, it may also lie partly on the causal pathway between parental asthma and offspring asthma phenotypes.
